# A Comparison between the Jenkins Porcelain Enamel and the High-Fusing Porcelain

**Published:** 1904-06-15

**Authors:** J. Q. Byram

**Affiliations:** Indianapolis, Ind.


THE
### DENTAL REGISTER

Vol. LVIII. JUNE 15, 1904. No. 6.

### A COMPARISON BETWEEN THE JENKINS PORCELAIN ENAMEL AND THE HIGH-FUSING PORCELAIN.

BY J. Q. BYRAM, D.D.S., INDIANAPOLIS, IND.

Read before the Ohio State Dental Society, December, 1903, and printed from advanced proofs by courtesy of "Dental Summary."

Many of those who engaged in the practice of dentistry before it was considered a profession expressed a desire to find a filling material that would satisfy the esthetic sense by approaching the color of the natural teeth. That desire has since found frequent expression, and experiments have been made with a number of materials; the two receiving the most attention being cement and porcelain. As the former has so far failed to fulfill more requirements than the latter, our best efforts have been directed to perfecting the use of porcelain rather than cement. The fact that its use is constantly increasing is evidence that a large number of dentists are becoming acquainted with its merits. "Porcelain as applied to the dental art, prior to 1885, was practically confined to the manufacture of teeth and a few cavity stoppers. About this time a method of constructing a metallic matrix representing the cavity and filling it with glass or porcelain was devised." By constant efforts of a few dentists, porcelain has been kept before the profession and now it is generally recognized as a material of merit for the restoration of lost tooth structure in those cases where metals are objectionable because of their lack of harmony with the color of the teeth.

Since the introduction of cohesive gold, no material has received the same consideration that porcelain has received in the last four years. Many of us are asked: Is porcelain to be added to the list of permanent filling materials, or is it a fad to insert porcelain inlays, and will this, like many other fads, pass away in a short time? It is true that we are sometimes carried away by certain fads, but if a fad has been an education to a higher appreciation of dental services, then it has been of some value. The gold crown fad has passed, but we have learned that badly-broken-down teeth can be preserved and made useful. Cataphoresis has taught us that a pulp can be anesthetized by the diffusion of certain drugs through the dentin to the pulp and pressure anesthesia is the result.

One of the elements of true dental art lies in disguising the artificiality of the restoration of lost tissue or organs, and he who is able to disguise his art is truly the artistic dentist. If the insertion of porcelain inlays has been carried to an extreme by some enthusiastic exponents, their enthusiasm has caused other dentists to become interested in porcelain and their esthetic sense has developed to a higher degree so that they now practice all dentistry in a more artistic manner.

To what extent porcelain will be used for the restoration of lost tooth structure depends largely on the dentist and his patients. Before porcelain can be used as a universal filling material, a large number of dentists will be compelled to develop the esthetic side of their natures. They cannot become artists if they are lacking in esthetic tastes and appreciation of the harmonies of nature. Much is said about educating the public to a higher appreciation of dentistry, but the tendency of the profession to commercialism prevents many artistic operations from being performed. The questions that so often confront us are: "What will it cost?" and "how long will it last?" The success of the operation is determined by the price and durability from such patients' point of view.

Some of the advantages of porcelain inlays are:

1. Filling can be inserted which only the expert may detect.

2. They are non-conductors of thermal changes.

3. The margins of cavities filled with porcelain are not readily attacked by caries.

4. The patient is relieved of the excruciating pain of adjusting rubber dam clamps for cavities extending beneath the gum.

5. The nervous strain on both patient and dentist is lessened.

6. Porcelain gives a better masticating surface than metal.

7. Busy patients need not spend so much time in the dental chair.

The disadvantages of porcelain are:

1. The friability of porcelain causes it to fracture readily.

2. It is impossible to bevel the cavity margins to protect the enamel.

3. It is difficult to match the color of the natural teeth.

4. The cement used as a retaining medium may cause a change of color in the tooth or inlay.

5. The cement will be dissolved, unless there is only a thin film used as a retaining medium.

Porcelain is not applicable for all forms of restoration and it should not be expected to entirely displace gold and amalgam as filling materials. When properly applied and used where indicated, it ranks first as a filling material. The indications of porcelain as a filling material may be classified first, according to the condition of the patient, and second, according to the class of cavities.

Under the first classification come the following:

1. In the teeth of nervous patients where it is almost impossible to prepare retentive forms of cavities for gold or amalgam fillings.

2. For those patients, either young or old, where the shock from the insertion of a large gold filling will almost cause nervous prostration.

3. For that class of patients who have a keen appreciation of artistic dentistry and who object to the conspicuousness of metallic fillings.

4. For those cases where the pericementum and alveolar process are diseased, which always exclude any material which requires a great deal of condensing.

5. In teeth where caries has progressed to such an extent that the pulp is almost involved, and if filled with a metal the irritation caused by thermal changes will cause death of the pulp.

The class of cavities where porcelain is indicated may be as follows:

1. All cavities on the labial and buccal surfaces of teeth.

2. All simple proximal cavities on incisors and cuspids.

3. Proximo-incisal cavities, if the cavities can be so prepared that the retentive resistance shall be greater than the stress.

4. Cavities involving all or a portion of the incisal edge.

5. Deep cavities on the occlusal surfaces or molars.

6. Proximo-occlusal cavities of bicuspids and molars, provided a large enough mass of porcelain can be used to withstand the force of mastication.

7. Cavities involving the entire occlusal surfaces of molars.

The contra-indications for porcelain inlays are:

1. In cavities where the stress will dislodge the filling or cause it to fracture.

2. In all cavities where it is impossible to construct a good matrix because of the inaccessibility of the cavity.

3. On incisors which are thin labio-lingually through the incisal third.

The construction of porcelain inlays involves four sets of manipulative principles:

1. The preparation of the cavity.
2. The construction of the matrix.
3. The fusing of the porcelain.
4. The setting and finishing of the inlay.

Each step presents a number of mechanical principles which if not properly observed will cause an imperfect filling. The failure of many inlays may be attributed to faulty manipulation, for too many beginners attempt the construction of inlays for the mouth before they have mastered the technique.

That many dentists fail to comprehend the principles by which inlays are retained is shown by their cavity preparation, and when asked how they expect the inlay to be retained, their answer is, "by the cement." This lack of comprehension may be largely due to the radical statements of some of the porcelain enthusiasts, some of which are as follows:*"For those bordering on nervous prostration and those high-strung, nervous temperaments for whom it is a physical impossibility to prepare a cavity even for a cement filling, to say nothing of gold, you can do permanent work with porcelain." "After the cement has completely crystallized, a thin porcelain filling in the occlusal surfaces of molars will have the full strength of the whole tooth to resist the masticating stress and is in no danger of fracture. * * * * Dentists have been brought up on the law of self-retentive form of cavity and interlocking form of filling and it is hard for them as inlay workers to break away from that law. There are inlay workers to-day who are working upon self-retentive forms of cavity formation, and grooving their inlays or baking into them platinum pins or loops to make them as near interlocking as possible. Inlays depend upon the law of close adaptation and the medium of completing the close adaptation crystallizing under pressure."

---

*Dental Review*, May, 1902

If such statements were true, there would be no need of other filling materials. But such as these have misled many skillful dentists and after a few failures they have decided that porcelain as a filling material is not practicable.

Cavities for inlays should be prepared with the same carefulness that should be used for other fillings. The opposite walls of simple cavities should be parallel and form right angles with the pulpal wall. In cavities where steps are used they should involve enough of the tooth structure to resist the force of mastication. It is better to cut the step through the middle third of the crowns to prevent the porcelain and cement from showing through the enamel on those incisors that are thin labio-lingually through the incisal third. Where pins are inserted to assist in retaining inlays, in teeth with vital pulps, they should extend far enough into the dentin to give necessary resistance, but not far enough to encroach upon their pulps. All frail enamel should be removed and the margins of all cavities should be smooth and in definite curves or straight lines. The margins should be so formed that there will be no short bevels to give frail edges of porcelain.

The best instruments for the preparation of cavities for inlays are flat-faced fissure and inlay burs, small Arkansas stones and excavators with short blades and their edges so formed that they will make all angles either right or obtuse and well defined.

There are three general methods of constructing matrices for cavities:

1. Swaging the foil over a negative or an impression of the cavity.
2. Swaging into a positive of the cavity.
3. Burnishing directly into the cavity.

In cavities involving only the labial or buccal or lingual surface or in simple proximal cavities, a matrix can be swaged over an impression and an inlay constructed which accurately fits the cavity. The method of swaging into a positive die of the cavity has some followers, but it hardly seems possible that a positive die can be obtained that will reproduce the margins of the cavity with that degree of accuracy that is required in inlay work. The method of burnishing the foil directly into the cavity has more advocates than either of the other methods, and many who have tried the swaging process have returned to this method.

A combination of the swaging and burnishing methods can be used successfully. The technic of constructing a matrix is as follows: Take an impression of the cavity in dental lac or cement, and then construct a positive die of the cavity by mixing a mass of cement and embedding the impression in it. Allow the cement to harden, then separate and invest the die in the ring of a swaging device. Coat the die with soap-stone, then place a piece of foil over it and swage with a water-bag or vulcanized rubber. Remove the matrix and cover the floor with porcelain and fuse. Now fit the matrix into the cavity and burnish the metal to the margins. The heat required to fuse the porcelain thoroughly anneals the metal. This method is especially good in large cavities, and has the advantage of conforming the foil to the cavity by the use of the die so that the final burnishing is made quite easy.

### THE FUSING OF PORCELAIN.

Porcelain is divided into high and low-fusing bodies. There is a difference of opinion regarding the relative merits of each form, and the ardent advocates of the one can see no good in the other. Both have their application and the dentist should no more confine himself to one form of porcelain than he should to one form of gold or alloy.

All high-fusing porcelains contain silica, kaolin, feldspar, a flux and the oxids of certain metals. Silica is the oxid of silicon; it is almost infusible and insoluble in all acids except hydrofluoric. It gives the fused porcelain a translucent appearance and adds strength. Kaolin is the silicate of aluminum and is a very refractory clay. This is added to porcelain to impart stability of form and it is this property which permits it to be molded and carved. Relatively large proportions of kaolin tend to give porcelain an opaque appearance. Feldspar is a double silicate of aluminum and potassium. "On account of its ready fusibility, it serves to agglutinate the more refractory ingredients, and when diffused throughout the mass imports a semi-translucent appearance." The flux is composed of some of the following: glass, the carbonates of sodium and potassium, calcined sodium borate, sodium and aluminum fluorid (cryolite). One or more of these materials is added to the silica or feldspar, or both, and are used to render the porcelain more fusible.

The basal ingredients of high-fusing porcelain may be used in a formula of low-fusing, but the fusing point is regulated by the proportion of flux added to the formula. The flux is incorporated with basal ingredients and the same heated until the flux agglutinates the refractory materials. The process of fusing and grinding is continued until all the ingredients are thoroughly mixed. The action of the flux on the refractory materials increases the fusibility and the porosity of the porcelain.

The Jenkins porcelain enamel is classed as a low-fusing body. It is prepared by a process of manufacture not in vogue in any other laboratory, and it has properties not possessed by any other low-fusing porcelain. It has density, hardness and strength equal to any of the high-fusing bodies. Its colors more nearly harmonize with the natural teeth. *Dr. Walter W. Bruck, of Breslau, Germany, says: "It has been claimed that the Jenkins material does not differ essentially in comparison from the glass powders previously in use, and that it melts over a Bunsen burner, which fact justifies the suspicion that it is nothing more than a glass compound. I have busied myself in these last years, not only with making fillings after the Jenkins system, but

---

*"The Method of Filling Teeth with Porcelain," translated from the German.

also have been interested in studying the composition and fusing point of this porcelain enamel and of other compounds of like nature. * * * * I had some of the porcelain mixtures, which have come most into use, analyzed in the chemical institute of the Breslau University, the result being that the Jenkins powder is known to be almost identical in composition—the variation being but slight, with the so-called 'high-fusing' porcelain as well as with the hard German porcelain tested by H. Segar, but not more exactly designated.''

The technic of preparing a cavity and constructing a matrix is the same for the Jenkins porcelain enamel as for the high-fusing porcelains. Either gold or platinum foil may be used for the matrix. On account of the pliability of gold, some of the advocates of the Jenkins enamel claim that its chief advantage is that it may be burnished into deep cavities, and better adapted to the cavity margins. It is true that gold is more pliable than platinum, but with the improved form burnished by the S. S. White Dental Mfg. Co., a matrix for the cavity of any depth can be constructed of platinum that will conform to its walls and have its cavity margins definitely defined. Doctor Jenkins recommends the use of No. 30 gold-foil for constructing matrices. Because of thinness of this gauge foil, the matrix tends to distortion upon removal from the cavity and to change form during the process of fusing the porcelain. Any material used for a matrix should be rigid enough to retain its shape upon removal from the cavity, or when heated to the fusing point of the porcelain.

A table compiled by Dr. W. A. Capon and Mr. J. F. Hammond gives the approximate fusing point of Jenkins' enamel 1544 F., Brewster's enamel 2047, Brewster's foundation 2210 and S. S. White's and Close's body at 2300. The low degree of heat required to fuse the Jenkins' enamel permits it to be fused in the flame of a gas or gasoline blowpipe. This is an advantage over high-fusing porcelains for those dentists who are unable to procure the electric current.

The shrinkage of the Jenkins enamel is greater than that of the high-fusing porcelains. The slight affinity of the porcelain, while fusing for the matrix, seems to increase the tendency of the mass to form a spheroid, leaving a space between the walls of the matrix and the porcelain. Because of the amount of shrinkage, it requires from four to six bakings for an inlay made of this enamel, where two or three would suffice for the same sized matrix filled with high-fusing porcelain. The time consumed in baking a small inlay of this enamel is less than that required for the high-fusing porcelain.

The Jenkins enamel is harder, denser and less brittle and will resist a greater stress than the high-fusing porcelains. The surface of the porcelain presents a less porous appearance when ground, so when inlays constructed of this material are ground, they can be given a high polish.

The tendency of the Jenkins enamel to form an amorphous mass in the matrix after the moisture has been absorbed is one of its disadvantages. This may be due to the lack of an ingredient in the compound to impart stability of form. It is more difficult to construct proximo-incisal and proximo-occlusal fillings of this material than of the high-fusing porcelains, because the properties of enamel prevent it from being readily carved.

Regarding the availability of the Jenkins porcelain enamel, we quote again from Dr. Walter W. Bruck: "The mistrust of new discoveries and methods is to a certain degree excusable, since it often occurs that quite useless innovations are extravagantly praised. It is certainly desirable that warnings founded on experience should be published in case of useless and worthless things."

"When Doctor Jenkins, whose standing as a practitioner I have no need to certify, made known his method after years of experimentations, he not only explained it theoretically in a manner worthy of admiration, but also exhibited it practically in the mouths of his patients with brilliant and almost never failing success. Such a discovery, thus announced, may be received at once with a degree of confidence. That in this case, confidence was completely justified, is proven by the many satisfactory trials of this method by German dentists and reported by them both verbally and in writing. So much the more astonishing is it that such really useful discoveries are often subjected to unreasonable criticism." Dr. W. H. Taggart says, "*The man who vigorously condemns a low-fusing body will have a lot to take back next year, and the low-fusing man who ridicules high-fusing bodies will have more to take back. There are possibilities in each one. * * * * Some of the most beautiful inlays I have ever made were made with the Jenkins bodies. * * * * There is no doubt in my mind, but that the Jenkins body has physical properties which make it suitable for use in certain cavities. The fact that it has not been used more than four or five years is no argument against it. We should strive to learn its possibilities."

A brief resume of the properties of the Jenkins porcelain enamel is as follows:

It is less brittle than the high-fusing porcelain.

It is fully as strong.

It more nearly imitates the appearance of the natural teeth.

Its density and hardness are greater than that of the high-fusing porcelains.

Its shrinkage is greater.

It is more difficult to restore contours with the Jenkins enamel.

It requires less time to construct simple inlays of this material.

It is a good material for dentists who are unable to obtain an electric current.

---

*Dental Review*, September, 1903.

